# Internet and *WeChat* used by patients with Crohn’s disease in China: a multi-center questionnaire survey

**DOI:** 10.1186/s12876-019-1011-3

**Published:** 2019-06-20

**Authors:** Qiao Yu, Liyi Xu, Lili Li, Min Zhi, Yubei Gu, Xinying Wang, Hong Guo, Yue Li, Yihong Fan, Bolin Yang, Meng Xue, Minfang Lv, Dingting Xu, Hanyun Zhang, Yan Li, Yongmao Song, Qun Deng, Xiaoxu Huang, Jing Zhong, Wen Hu, Yimiao Zhu, Xiaoying Wang, Jianting Cai, Yan Chen

**Affiliations:** 1 Center for Inflammatory Bowel Diseases, Department of Gastroenterology, The Second Affiliated Hospital, School of Medicine, Zhejiang University, No. 88, Jiefang Road, Hangzhou, 310009 Zhejiang Province China; 2 Department of Gastroenterology, the Sixth Affiliated Hospital of Sun Yat-sen University, Guangzhou, Guangdong Province China; 3 Department of Gastroenterology, Rui Jin Hospital, Affiliated to Shanghai Jiao Tong University, School of Medicine, Shanghai, China; 4 Department of Gastroenterology, Zhujiang Hospital, Southern Medical University, Guangzhou, Guangdong Province China; 5 Department of Gastroenterology, Xinqiao Hospital, Army Medical University, Chongqing, China; 6 Department of Gastroenterology, Peking Union Medical College Hospital, Beijing, China; 7 Department of Gastroenterology, Zhejiang Provincial Hospital of Traditional Chinese Medicine, Hangzhou, Zhejiang Province China; 8 Department of Colorectal Surgery, the Affiliated Hospital of Nanjing University of Chinese Medicine, Nanjing, Jiangsu Province China; 9 Department of Oncology Surgery, the Second Affiliated Hospital, Zhejiang University School of Medicine, Hangzhou, Zhejiang Province China; 10 Department of Nutrition, the Second Affiliated Hospital, Zhejiang University School of Medicine, Hangzhou, Zhejiang Province China

**Keywords:** Multicentre, Questionnaire survey, Internet, Social media, Inflammatory bowel disease, Crohn’s disease

## Abstract

**Background:**

Currently, *WeChat* is widely used in disease education for patients with Crohn’s disease (CD) in China. It is beneficial for the patients to actively engage in their disease management.

**Methods:**

In this study, we examined the source and expectations of disease information for Chinese CD patients, analysing the content of popular *WeChat* public accounts and their potential association with medication adherence.

**Results:**

Between November 24th, 2017 and April 10th, 2018, online questionnaires were sent to CD patients from eight different large urban hospitals in China. In all, 436 patients with CD were surveyed, and 342 patients responded. Patients most frequently visited *Baidu* (65%)*, WeChat* (61%) and medical websites such as *Haodaifu* (35%) when searching for IBD-related information. Among ten *WeChat* IBD public accounts, the China Crohn’s and Colitis Foundation (CCCF) (73%), “*IBD Academic Officer*” (21%) and “*IBD in love*” (21%) were the most popular. CD patients were most interested in information from the internet about diet and day-to-day health-related living with IBD (83%), an introduction to the disease (80%), and medication advances and side effects (80%). The correlation between the information provided by the top five *WeChat* public accounts and patients’ expectations was low. Additionally, most patients (64%) had greater confidence in overcoming the disease after learning about CD through their internet searches. Medical adherence was also related to internet access and income (*p* < 0.05).

**Conclusions:**

*WeChat* has become a major source of information for IBD education in China, but the content of *WeChat* didn’t fully meet patients’ expectations. Therefore, future initiatives should aim to provide high-quality information that based on patients’ demands.

## Background

Inflammatory bowel disease (IBD), which mainly includes ulcerative colitis (UC) and Crohn’s disease (CD), is a non-specific chronic inflammation of the intestinal tract. The incidence of IBD is rising in China, and the tendency for lifelong recurrence decreases the quality of life of IBD patients, especially those with severe disease [1, 2]. Effective health education is helpful for the understanding and management of IBD.

Today, the internet and social media are widely used to acquire information. *WeChat*, one of the most popular social media sites in China, has over 1 billion active users over the world [3], far more than Facebook. *WeChat* supports not only messaging and free calling but also all kinds of voice, photos, videos and articles, and *WeChat* is deemed as an appropriate platform for patient education [4, 5]. Many new public online platforms for IBD have emerged in China.

Data from Western countries have proven that patients with better IBD knowledge have higher medication adherence and better outcomes [6, 7]. Current data showed that internet access for IBD patients was 81%, with 54% using the internet to obtain IBD-related information [8]. Up to 55% of IBD patients were willing to receive education through social media or organizations such as the Crohn’s and Colitis Foundation of America (CCFA) [9, 10].

The China Crohn’s and Colitis Foundation (CCCF) is a nonprofit, volunteer-driven organization dedicated to improving the quality of life of IBD patients in China through education and training [11]. CCCF volunteers, who include medical staff, patients, family members of patients and social volunteers, are a group of people full of love and faith dedicated to helping IBD patients [12]. These volunteers share their experience with other patients and help to organize various CCCF activities. However, the internet usage and attitudes towards the internet of CD patients and CCCF volunteers in China remain unclear.

This survey was performed to study the use of the internet and social media by CD patients in China, to compare the most popular *WeChat* CD public accounts and to analyse their impact of the internet and social media on the medication adherence of IBD patients.

## Methods

### Participants

This study was performed in the IBD centres in eight large urban hospitals in 6 cities (Beijing, Shanghai, Hangzhou, Chongqing, Nanjing and Guangzhou) in relatively economically developed provinces within mainland China. Informed consent was received from all patients who completed the questionnaire. Incidental outpatients or in-hospital IBD cases diagnosed with CD between November 24th, 2017 and April 10th, 2018 were invited to complete the questionnaire online. Patients were excluded if they had difficulty in reading or understanding the information or had no internet access. The online Questionnaire app in Chinese was used to collect primary and sample information for this study.

### Survey instruments

The questionnaire was designed by IBD physicians and revised by several senior doctors from each hospital. We used this questionnaire to sample 20 patients based on the inclusion criteria and analysed the questionnaire’s validity. The final questionnaire was approved by all the participating organizations. The questionnaire content included 4 sections with open and close-ended questions: 1) social demographic data and patients’ disease status, 2) IBD-related information sources and patients’ preferences, 3) patients’ expectations for online education, and 4) network influence and medication adherence. The information on medication adherence was examined using Morisky Medication Adherence Scale (MMAS)-8 scores [13]. Licensure agreement for the use of MMAS was available from Donald E. Morisky, ScD, ScM, MSPH, Professor, Department of Community Health Sciences, UCLA School of Public Health.

### Statistical analysis

The preliminary data were summarized using SPSS version 23.0. The qualitative data were expressed as percentages, and the quantitative data such as the MMAS scores were calculated and analysed using the mean and standard deviation. The differences between the groups were assessed using the Student’s t test and single factor analysis of variance. Multivariate analysis was used to determine the factors influencing the patients’ medication adherence. The Kappa value was used to evaluate the consistency between provided and expectation information of *WeChat*. A *p* value of 0.05 or less was defined as statistically significant.

## Results

### Demographics of patients

As shown in Table 1, 342 valid questionnaires from patients diagnosed with CD were used for the analyses. The median age of the respondents was 41 years; 60% of the patients were male, and 15% were CCCF volunteers. The patients went to at least 3 different hospitals because of either abdominal pain or diarrhoeal symptoms, but on average, only one out of the three hospitals had a specialized IBD clinic. The income of most patients was less than 10,000 RMB per month (79%). The most common educational levels of the patients participating in this survey were college (59%) and high school or technical school (36%).

**Table 1:** Participant characteristics (*n* = 342)

Demographic characteristics	Number (%)
Gender	
Male	205 (60)
Female	137 (40)
Age (y)	41 [37,45] ^a^
Monthly income(¥)	
Less than 5000	128 (37)
5001–9999	145 (42)
10,000–19,999	40 (12)
More than 20,000	29 (9)
Education	
Illiteracy	1 (0.3)
Primary school	10 (3)
High school or technical school	122 (36)
College	201 (59)
Postgraduate education	8 (2)
CCCF volunteers	
Yes	50 (15)
No	292 (85)

*CCCF* The China Crohn’s & Colitis Foundation

^a^Average age and age ranges

### Sources of disease-related information

**Fig. 1:**
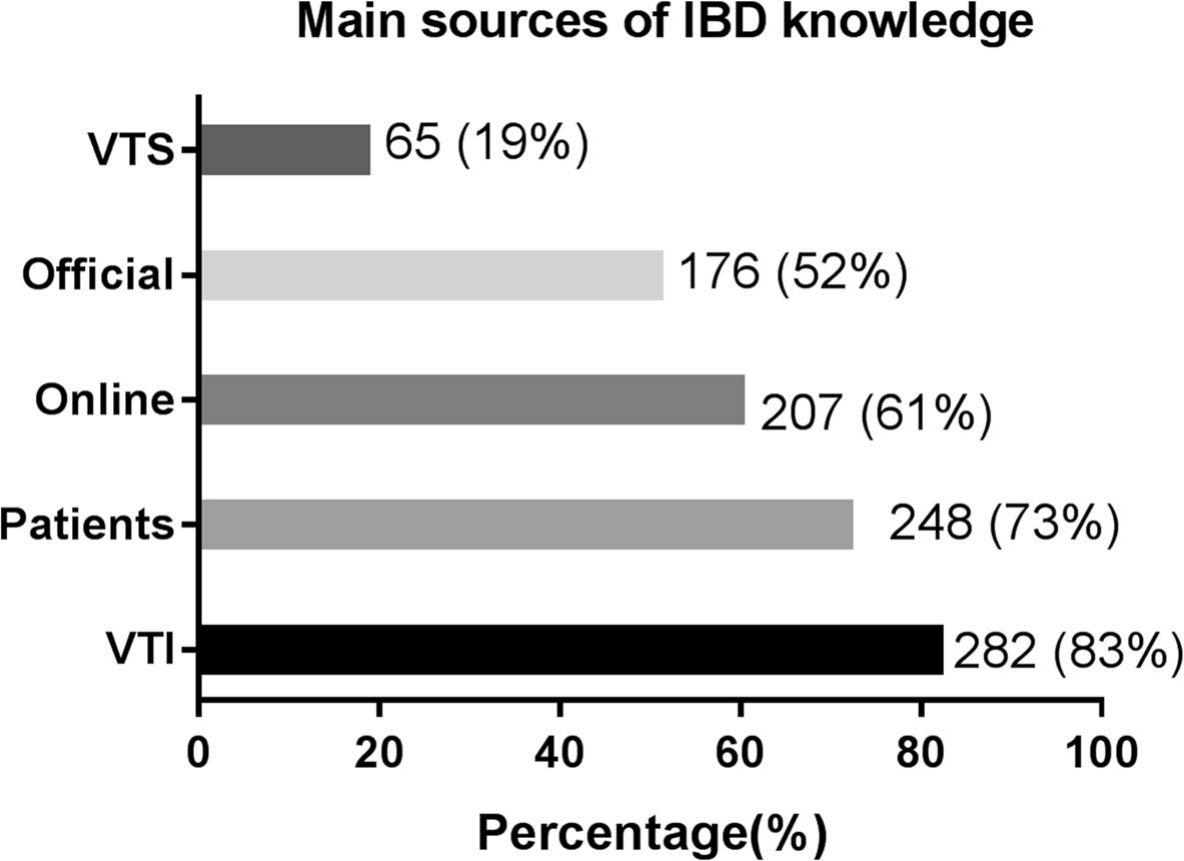
Main sources of IBD knowledge. VTS: verbal teaching from surgeons; Official: Official brochures or materials from hospitals; Online: Online searching/consultation by themselves; Patients: Communication with other patients with IBD; VTI: Verbal teaching from IBD specialists

**Fig. 2:**
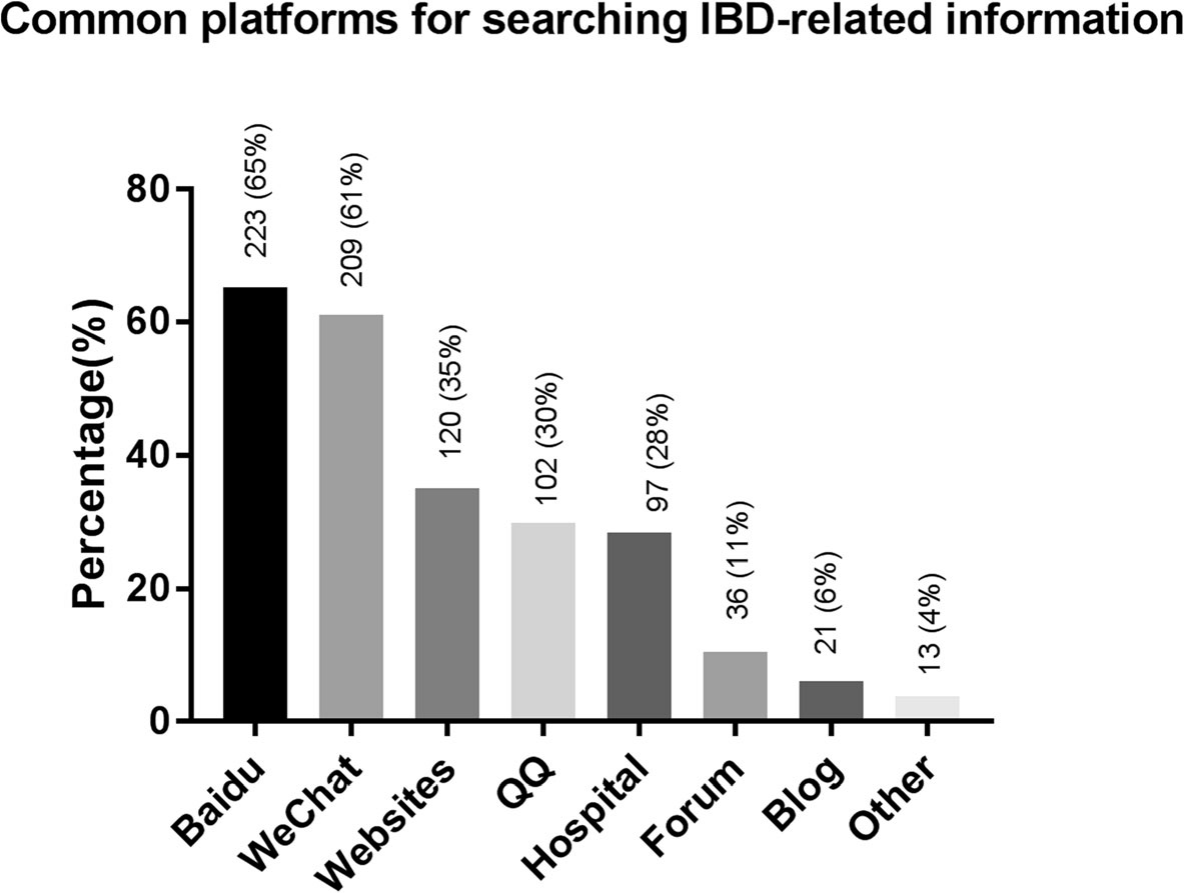
Common platforms for searching IBD-related information

Today, patients have access to information from a variety of sources. In our study, the most commonly used sources of information for CD patients were verbal teaching by IBD physicians (83%), communication with other patients (73%), online searches (61%), and official brochures or materials from hospitals (52%) (Fig. 1). Currently, amongst all the internet and social media sites in China, the most frequently used by CD patients were the *Baidu* search engine (65%), official *WeChat* accounts (61%), medical websites such as *Haodaifu* (35%), and QQ group (30%) (Fig. 2).

When asked about what patients expected to know about IBD (Table 2), 282 patients (83%) wanted suggestions about diet and living with IBD, 274 patients (80%) wanted general introductory information such as the aetiology, and clinical features of IBD and life expectancy of patients with IBD, and 274 patients (80%) were interested in the latest developments in treatment and the side effects of drugs. Among the respondents, only 130 (38%) of the patients were willing to pay for online consultations.

**Table 2:** Patients’ expectation of information sources

Expectation items	Number (%)
Expected sources to gain IBD knowledge	
Verbal teaching from IBD physicians	274 (80)
Official brochures or materials from hospitals	261 (76)
Videos from doctors	236 (69)
Communication with other patients	208 (61)
Referring to professional books	127 (37)
Videos recorded by patients	77 (23)
Internet searching by themselves	66 (19)
Others	5 (2)
Information expectations for IBD patients	
Notices in diet and daily life	282 (83)
Disease general introduction	274 (80)
Medication advances and side effects	274 (80)
The prognosis and influencing factors	204 (60)
Policy about subsidies and discounts for treatment	198 (58)
Relevant knowledge about surgery	164 (48)
Stories about defeating the disease	141 (41)
Communication with other patients through social media	141 (41)
Willingness to pay for online consultations of IBD	
Yes	130 (38)
No	51 (15)
Depends on the expenses	161 (47)

### Comparison of the five most popular official *WeChat* accounts

**Fig. 3:**
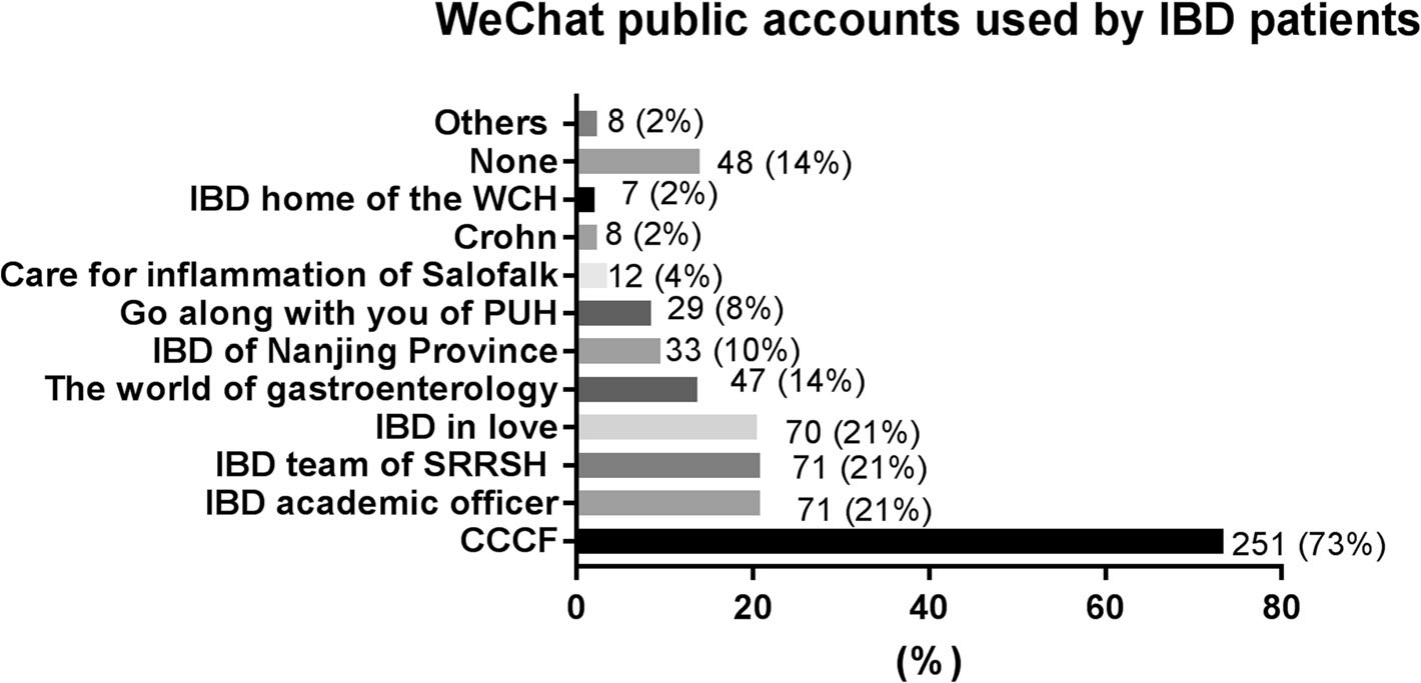
Wechat public accounts used by CD patients. WCH: West China Hospital; PUH: Peking Union Hospital; SRRSH: Sir run run Shaw hospital

*WeChat* public accounts have become an irreplaceable way for current healthcare news and practical knowledge for IBD patients. Therefore, we next focused on the most popular *WeChat* public accounts. As shown in Fig. 3, the top five most popular official *WeChat* accounts were CCCF (73%), “*IBD academic officer*” (21%), “*The IBD team of Sir Run Run Shaw Hospital*” of Zhejiang University (21%), “*IBD in love*” of the sixth affiliated hospital of Sun Yat-sen University (1%) and “*The world of gastroenterology*” (14%).

We further investigated the details of these top five *WeChat* public accounts, as shown in Table 3, and gathered data on their articles published between April 2017 and April 2018. Three of the five *WeChat* public accounts were founded by large urban hospitals whereas two were founded by personal volunteers. The number of articles published in the surveyed year varied from 59 to 393. In the five *WeChat* accounts, 96, 85, 97, 88, and 78% of the articles focused on CD. The update time, reading number, comments and likes for each article varied greatly. We summarized and classified the article topics into 12 categories, 8 of which matched the topics that patients expected to know about IBD in Table 2. The consistency between patients’ expectations and the actual contents of article topics was also analysed. The results showed that the articles published on the of *WeChat* public accounts largely did not meet patients’ expectations. The information given on “*The IBD team of Sir Run Run Shaw Hospital”* showed the highest match with patients’ expectations.

**Table 3:** Information of the five most popular *WeChat* official accounts (from August 16, 2017 to August 16, 2018)

Official *WeChat* accounts	CCCF	IBD in Love	The IBD team of Sir run run Shaw hospital	IBD Academic Officer	The World of Gastroenterology
Organization	The China Crohn’s & Colitis Foundation	The 6th hospital of Sun Yat-sen university	Sir run run Shaw hospital of Zhejiang University	Technology company and medicine related person	Technology company and medicine related person
Duration since setting up	55 months	47 months	46 months	16 months	48 months
Total number of articles	260	124	59	393	147
Update frequency (days) ^a^	1.40	2.94	6.19	0.93	2.48
Average reading number for each article	1479 ± 1174	1176 ± 1060	1419 ± 849	1092 ± 665	1110 ± 880
Highest reading number	10,810	4885	5626	6084	4234
Comments	6.51 ± 10.38	1.60 ± 3.58	1.49 ± 5.41	1.41 ± 1.92	0.32 ± 1.06
Likes	23.07 ± 20.85	13.90 ± 12.36	24.60 ± 31.00	12.58 ± 7.12	6.38 ± 6.82
Focus on CD	249 (96%)	106 (85%)	57 (97%)	346 (88%)	115 (78%)
Topic types [number (percentage%)]					
1.Notices in diet and daily life	20 (8%)	12 (10%)	4 (7%)	13 (3%)	8 (5%)
2.General disease introduction	28 (11%)	4 (3%)	7 (12%)	85 (22%)	77 (52%)
3.Medication advances and side effects	10 (4%)	11 (9%)	5 (8%)	109 (28%)	11 (7%)
4.The prognosis and influencing factors	9 (3%)	6 (5%)	4 (7%)	70 (18%)	7 (5%)
5.Policy about subsidies and discounts for treatment	0 (0%)	1 (0.8%)	1 (2%)	3 (0.8%)	0 (0%)
6.Relevant knowledge about surgery	7 (3%)	3 (2%)	4 (7%)	29 (7%)	6 (4%))
7.Communication with other patients	20 (8%)	15 (12%)	0 (0%)	0 (0%)	0 (0%)
8.Stories about defeating the disease	13 (5%)	6 (5%)	8 (14%)	0 (0%)	0 (0%)
9.Consensus and research	54 (21%)	17 (14%)	4 (7%)	66 (17%)	38 (26%)
10.Education activities for patients	80 (31%)	32 (26%)	12 (20%)	7 (2%)	0 (0%)
11.Clinical guidance	5 (2%)	6 (5%)	9 (15%)	1 (0.3%)	0 (0%)
12.Others	7 (3%)	4 (3%)	0 (0%)	3 (1%)	0 (0%)
Kappa value ^b^	−0.143	0.143	0.286	0.259	0.000

^a^Update frequency means average days to update an article paper

^b^The kappa value represents consistency between the topic types of the five most popular *WeChat* official accounts and information expectations of CD patients

### The impact of the internet and social media use on patients’ medical behaviour

Nearly 70% (287/476) of the patients believed that the information they gained from the internet affected their choices of doctors and hospitals for medical service. Moreover, most of the patients (303/476, 63%) had greater confidence in managing their disease after learning about related knowledge through the internet, with only 14% (65/476) of patients feeling more anxious and pessimistic about their treatment from the knowledge gained online. In total, 47/476 patients, (10%) thought that the internet had no impact on their attitude to their disease. Most of the patients would not question their doctors’ decision based on information gained on the internet (413/476, 87%).

Multivariate analyses were conducted to explore the relationship between patients’ internet searches and number of visits to doctors. We found that patients who frequently searched the internet for IBD knowledge went to their follow-up hospital appointments more regularly in compared with those patients who did not like using the internet (*p* = 0.046). Analysis of the MMAS scores (Table 4) showed that internet access and income were predictors of better disease compliance (*p* < 0.05). However, no correlation was found between willingness to join an IBD-related group chat and medication adherence.

**Table 4:** Multivariate analysis of risk factors associated with medication adherence

Factors	95% Confidence interval	Beta coefficient
**Income**	**0.05–0.5**	**0.252**
Gender	−0.5-0.2	−0.165
Age	−0.02-0.02	0.002
Education	−0.2-0.1	−0.038
CCCF volunteers	−0.1-1.0	0.455
**Main ways of internet access**	**0.03–0.7**	**0.347**
Willing to join in IBD-related group chat	−0.4-0.02	−0.183
Main sources of disease knowledge	−0.1-0.3	0.129
The use of *WeChat* public accounts	−0.1-0.6	0.228
Intestinal surgery	−0.4-0.4	0.013

The bold figures are significant factors with medication adherence, and beta coefficient represents contribution value

## Discussion

This research is a multicentre survey investigating the association between information sources for CD patients and popular social media sites in China. We compared the top five commonly used *WeChat* public accounts and their consistency with patients’ desired information and evaluated the impact on of these sites on medication adherence, providing a reference for improving further online education for IBD.

Over time, the traditional doctor-patient passive education model has gradually transformed into a model of active learning by patients themselves. A Spanish study found that 84% of patients showed an interest in IBD websites supported by the physicians of their referral centre [14]. Studies from Ireland and the UK showed that IBD patients preferred getting face-to-face information from specialist doctors or nurses rather than the internet [15, 16]. In our study, verbal teaching from gastroenterologists, communication with other IBD patients and online searches were the top three information sources for IBD patients, and this finding is consistent with those from previous studies. Moreover, CCFA was the most frequently used IBD-specific website in USA [9], and their official website (http://www.ccfa.org) ranked first in a study on the quality of IBD-related websites [16]. Google, Facebook and YouTube were the most popular search engines in USA [17, 18]. Compared with Western countries, we found that in China, *Baidu* and *WeChat* public accounts and medical related apps such as *Haodaifu* were the most common platforms scanned. On *WeChat*, CCCF, “*IBD Academic Officer*” and “*IBD team of Sir Run Run Shaw Hospital*” were the three most popular accounts among ten CD-specific *WeChat* public accounts. The success of these *WeChat* public accounts may be due to several reasons. First, “CCCF” is a non-profit, volunteer-driven organization aimed at helping patients affected by IBD through various education activities in China [11]. Second, the CD-specific *WeChat* accounts provided comprehensive information about IBD, mainly focusing on demands and caring of IBD patients. Lastly, the *WeChat* public accounts frequently updated their sites with new articles, thus attracting frequent readers’ attention.

What information do IBD patients desire? A study from Spain suggested that IBD patients expected to be fully informed about their social and work rights, cancer and mortality risk and research trials [19]. Another study in Ireland showed that IBD patients were most eager to obtain knowledge on medication, ‘what to expect in the future’, living with IBD, and diet [15]. In addition, a study in Canada found that long-term prognosis and guidance on diet, topics that were regarded as important, were the least adequately addressed [20]. In our study, consistent with results from other countries, advice on daily diet and exercises; the aetiology, clinical features and life expectancy of patients with IBD; and the latest developments in medicine were the top three preferences for CD patients in China.

As *WeChat* is the main way for patients to obtain information, we assessed whether these official accounts provided the required knowledge. It was observed that the top five *WeChat* public accounts concentrated on different aspects. CCCF, “*love in IBD*” and “*The IBD team of SRRSH*” concentrated on social activities and patient education, while “*IBD Academic Officer*” and “*The World of Gastroenterology*” focused mostly on medication-related knowledge and general disease introduction. We found only a weak correlation between the expectations of patients and the information. Moreover, very few articles presented content such as subsidies and discounts for treatment, which 60% of patients wanted to learn more about that. Therefore, the medical *WeChat* public accounts should ascertain the needs of patients and provide them with genuinely useful information.

The internet and social media sites not only gave guidance on medical treatment but also focused on psychological treatment and provided encouragement for patients. Our data showed that the choice of hospitals or doctors could change after internet searching. The searches allowed patients to learn more about professional IBD centres in different hospitals and choose more suitable treatment options. Reports from the UK revealed that frequent use of official information websites was associated with better disease-related knowledge and lower anxiety levels [21]. Our study demonstrated that most patients felt more confident in managing their disease after internet searching. The patients gained empathy and energy from other patients’ stories and experience which were very beneficial in managing their disease. The internet could help patients learn more about their disease and encourage more timely follow ups. However, a larger clinical data sample and more prognostic data analyses are needed to draw a more definitive conclusion.

Although previous studies have shown that patients with greater knowledge have better disease coping strategies, there has been no previous information on the relationship between knowledge and treatment adherence [7]. Our results showed that patients with higher incomes and greater access to the internet had better medication adherence. This outcome may be due to better disease understanding ability in high-income populations. Moreover, patients who frequently searched IBD information online had more timely follow-ups for their IBD clinic or hospitalization. However, there was no correlation with specific *WeChat* accounts or other internet factors. On one hand, more time may be needed for the knowledge gained online to inspire a change in attitudes and behaviour. On the other, medication adherence could be influenced by different confounding factors, and the resources available to the IBD network were not effective enough to improve medical compliance. We would therefore recommend a better combination and balance of online and offline IBD education activities.

There are several limitations in this study. First, although we surveyed a large number of out and inpatients from eight hospitals in different areas, the sample may not be representative of all Chinese CD patients. Selection bias was inevitable which would make our study less persuasive. Second, our study did not include patients with no internet access, although these patients are potential network users and their information sources and expectations would add valuable information. Further studies are needed to focus on such patients.

## Conclusion

Internet and social media sites such as *WeChat* have become important sources of information for IBD education in China. However, the information provided in current *WeChat* public accounts do not fully meet patients’ expectations. Future initiatives should aim to provide high-quality information that based on patients’ demands and establish a personalized and effective web-based health care system.

## Data Availability

The datasets generated and/or analyzed during the current study are not publicly available due individual privacy but are available from the corresponding author on reasonable request.

## Abbreviations

CCCF: The China Crohn’s and Colitis Foundation
CCFA: Crohn’s Colitis Foundation of America
CD: Crohn’s disease
IBD: Inflammatory bowel disease
UC: Ulcerative colitis
